# Comparison of the Diagnostic Performance of qPCR, Sanger Sequencing, and Whole-Genome Sequencing in Determining Clarithromycin and Levofloxacin Resistance in *Helicobacter pylori*

**DOI:** 10.3389/fcimb.2020.596371

**Published:** 2020-12-17

**Authors:** Konrad Egli, Karoline Wagner, Peter M Keller, Lorenz Risch, Martin Risch, Thomas Bodmer

**Affiliations:** ^1^ Labormedizinisches zentrum Dr Risch, Buchs, Switzerland; ^2^ Institute of Medical Microbiology, University of Zurich, Zurich, Switzerland; ^3^ Laboratory Medicine, University Hospital of Basel, Basel, Switzerland; ^4^ Institute for Infectious Diseases, University of Bern, Bern, Switzerland; ^5^ Private University of the Principality of Liechtenstein, Triesen, Liechtenstein

**Keywords:** *Helicobacter pylori*, direct detection in gastric biopsies, (quantitative) real-time PCR, phenotypic drug resistance, 23S rDNA gene, *gyrA*, hybridization probes

## Abstract

*Helicobacter pylori* antibiotic resistance is increasing worldwide, emphasizing the urgent need for more rapid resistance detection prior to the administration of *H. pylori* eradication regimens. Macrolides and fluoroquinolones are widely used to treat *H. pylori*. In this study, we aimed to compare the diagnostic performance of A) 23SrDNA qPCR (with melting curve analysis) and an in-house developed *gyrA* qPCR followed by Sanger sequencing with a commercial IVD-marked hybridization probe assay (for 23SrDNA and *gyrA*) using 142 gastric biopsies (skipping culturing) and B) the same two qPCR for 23SrDNA and *gyrA* (including Sanger sequencing) with whole-genome sequencing (WGS) and phenotypic characterization of clarithromycin and levofloxacin resistance using 76 cultured isolates. The sensitivity of both qPCRs was 100% compared to that of the commercial IVD-marked hybridization probe assay for the detection of *H. pylori* in gastric biopsies (without resistance testing). The specificity of the qPCR *gyrA* followed by Sanger sequencing was 100%, indicating that the best sequence identity was always *H. pylori*. The results show good agreement between molecular tests, especially between qPCR (inclusive Sanger sequencing) and WGS. Discrepancies (concerning mutated or wild type of positive *H. pylori* gastric biopsies) were observed between Sanger sequencing of the *gyrA* gene and the corresponding commercial hybridization probe assay, mostly because the high sequence diversity of the *gyrA* gene even at positions adjacent to the relevant codons of 87 and 91 interfered with obtaining correct results from the hybridization probe assay. Interestingly, we found several mixed sequences, indicating mixed populations in the gastric biopsies (direct detection without culturing). There was a high percentage of both levofloxacin and clarithromycin resistance in gastric biopsies (both between 22% and 29%, direct detection in gastric biopsies). Therefore, we recommend analyzing both targets in parallel. We confirmed that phenotypic resistance is highly correlated with the associated mutations. We concluded that the two qPCR followed by Sanger sequencing of the *gyrA* gene is a fast, cost-effective and comprehensive method for resistance testing of *H. pylori* directly in gastric biopsies.

## Introduction

*Helicobacter pylori* is a gram-negative bacterium that infects approximately half of the human population worldwide. *H. pylori* infection is a major determinator in the etiology of various gastrointestinal diseases, including chronic active gastritis, peptic or duodenal ulcers, gastric adenocarcinoma, and mucosa-associated tissue lymphoma. Clarithromycin, in combination with metronidazole or amoxicillin and a proton pump inhibitor (Gisbert et al., 2000; Malfertheiner et al., 2017; Marques et al., 2020), has been the key drug in the first-line *H. pylori* eradication regimen for more than a decade. However, the widespread use of clarithromycin has led to an increase in clarithromycin-resistant *H. pylori*, diminishing the effectiveness of conventional first-line treatment regimens and increasing treatment failures due to drug-resistant *H. pylori* (Wueppenhorst et al., 2009; Buzás, 2010; Thung et al., 2016). An increasing trend of clarithromycin resistance in *H. pylori* can be observed worldwide and specifically in European countries, with an overall primary clarithromycin resistance rate of 17%–21% (de Francesco et al., 2010; Dolak et al., 2017; Bilgilier et al., 2018). Although levofloxacin resistance has not been studied as extensively, there is also a trend towards increasing primary and secondary levofloxacin resistance in *H. pylori* (Hug and Rossi, 2001; Mandell et al., 2007). With rising antibiotic resistance rates, the usefulness of the currently applied “test-and-treat” strategy is questionable, emphasizing the need for rapid and accurate diagnostics to assess drug resistance in *H. pylori* prior to the administration of antibiotics.

Clarithromycin resistance in *H. pylori* is mainly attributable to three single point mutations (A2146C, A2146G, and A2147G) in the 23S rDNA (Zullo et al., 2007; Boyanova et al., 2010; Mégraud, 2012; Saracino et al., 2012), while mutations in the gyrase gene (*gyrA*; mainly at codons 87 and 91) are associated with levofloxacin resistance (Boyanova et al., 2010). Despite advances in *H. pylori* diagnostics, culture-based phenotypic drug susceptibility testing (DST) is still the most commonly applied method. However, the successful isolation and cultivation of *H. pylori* from gastric biopsy specimens is a challenging task hampered by a number of technical factors (e.g., quality of the clinical specimen, occurrence of microbial commensal flora, inappropriate transport conditions) (Cuadrado-Lavín et al., 2012). Furthermore, culture-based methods require highly trained laboratory personnel and take up to 14 days to obtain results. Rapid and accurate molecular methods that can detect *H. pylori* and assess its drug resistance are becoming increasingly important in diagnostics with rising antibiotic resistance.

Additionally, whole-genome sequencing (WGS) has been successfully employed for the detection of mutations that result in phenotypic drug resistance in *H. pylori* (Boyanova et al., 2010). With advances in sequencing technology (e.g., affordable instrument pricing, user friendly and simple workflows, standardization of protocols, and reagents, reasonable per sample costs), diagnostic laboratories can sequence culture isolates in a clinically relevant timeframe (24 to 48 h), providing a comprehensive view of the genotype and relevant drug resistance mutations of a bacterial isolate (Redondo et al., 2018; Lauener et al., 2019). However, a major remaining limitation of WGS is that it is still mostly performed on culture isolates, as the application of metagenomic approaches directly to gastric biopsies are hampered by low bacterial DNA content and high human DNA background (especially for DNA extraction methods without the depletion of human DNA).

To date, there are only a few commercially available molecular assays for the detection of mutations in the 23SrDNA (these tests are predominant) (e.g., LightMix Modular Helicobacter 23S rDNA, Genotype HelicoDR) and *gyrA* genes, and some in-house developed assays have been published (Schabereiter-Gurtner et al., 2004; Cambau et al., 2009; Bilgilier et al., 2018; Lauener et al., 2019; Makristathis et al., 2019; Jehanne et al., 2020). Specifically, quantitative real-time PCR assays (qPCR) combined with melting curve analysis enable the timely assessment of *H. pylori* and its drug resistance specimens (Schabereiter-Gurtner et al., 2004; Gazi et al., 2013; Bénéjat et al., 2018, Redondo et al., 2018) however, published data are limited to 23SrDNA. Design of a qPCR for *gyrA* remains a complex challenge due to the high sequence variation of *H. pylori* (Ailloud et al., 2019; Estibariz et al., 2019). To date and to our knowledge, only Genotype HelicoDR (Cambau et al., 2009) is used as a commercial test (with CE-IVD label) for the combined detection of different mutations of 23SrDNA and *gyrA*. However, this commercial kit is based on a time-consuming non-realtime PCR followed by hybridization which includes many incubation steps (Jehanne et al., 2020). Additionally, hybridization probes based analysis of *gyrA* gene (which have to differentiate between 1 bp sequence variation but only at relevant codon 87 and 91) conflicts with the above-mentioned extraordinary genetic diversity (Ailloud et al., 2019; Estibariz et al., 2019).

The goals of this study are a comprehensive comparison of different methods (culturing and molecular) in order to apply qPCR for a timely and sensitive susceptibility testing of clarithromycin as well as levofloxacin in parallel directly from gastric biopsy without culturing (which is hampered by different disadvantages). Especially for qPCR of *gyrA*, insufficient data are available (also for cultured isolates). Additionally, having up-to-date data from central Europe of the direct molecular susceptibility testing in gastric biopsies reveals a rather high (both between 22% and 29%) percentage of resistance in clarithromycin as well as levofloxacin resistance which might be due to selection of suboptimal antibiotic treatment of patient (Kenyon, 2019).

## Materials and Methods

### Clinical *H. pylori* Isolates and *H. pylori* Culture

This study was conducted using a set of 76 *H. pylori* strains from the bacterial strain collection of the Institute of Medical Microbiology (IMM), University of Zurich. The *H. pylori* strains were isolated between 2013 and 2017 from gastric biopsy specimens that were sent to the IMM for culture-based phenotypic DST. For this study, *H. pylori* strains were intentionally selected based on their antimicrobial resistance phenotype and are therefore, not representative of *H. pylori* primary antibiotic resistance epidemiology in Switzerland. After thawing, the strains were incubated on in-house produced Brucella agar plates (with 5% horse blood) for 3 days at 37°C under microaerobic conditions (90% N_2_, 5% CO_2_, 5% O_2_) using a gas generator (CampyGen, Thermo Scientific, Waltham, MA, USA). After 3 days, *H. pylori* isolates were subcultured on Brucella agar plates to obtain sufficient biomass for the E Test^®^ (bioMérieux, Marcy l’Etoile, France) and to perform DNA extraction for WGS.

### Phenotypic DST by E-Test^®^

*H. pylori* cultures were adjusted to a McFarland standard of 3 (Yuen et al., 2005). A McFarland of 3 was chosen as *H. pylori* is a fastidious bacterial pathogen, and sufficient growth is required on the Müller Hinton fastidious (MHF) agar plates in order to evaluate the E-Test results. In the diagnostic laboratory, previously published protocol for phenotypic susceptibility testing on fastidious organisms were considered (King, 2001). Phenotypic DST was performed on MHF agar plates containing 5% horse blood (bioMérieux) using the following E-Tests^®^ (bioMérieux): clarithromycin (0.016–256 mg/L) and levofloxacin (0.016–32 mg/L). Agar plates were incubated under microaerobic conditions at 37°C for 3 days. Subsequently, MICs were determined using a light microscope (Leica M80, Leica Microsystems, Heerbrugg, Switzerland). Susceptibility interpretation was performed according to the guidelines of the European Committee on Antimicrobial Susceptibility Testing (Redondo et al., 2018).

### DNA Extraction, qPCR, and Melting Curve Analysis

DNA was extracted from unmodified (i.e., without fixation or embedment) gastric biopsy specimens (collected 2018) using the QIAamp DNA Mini Kit (order number 51304, Qiagen, Hilden, Germany) with proteinase K digestion, according to the manufacturer’s recommendations. Gastric biopsies had a diameter of 1–3 mm and duration of proteinase K incubation was at least 1h for complete dissolution. The clinical specimens were from the clients (e.g., gastroenterologist) of Dr. Risch diagnostic laboratory without any selections. DNA was extracted from *H. pylori* isolates with the DNeasy^®^ UltraClean^®^ Microbial Kit (Qiagen, Hilden, Germany), following the manufacturer’s recommendations.

For qPCR amplification of 23S rDNA, extracted DNA was added to a mixture consisting of Roche PCR-grade water (Roche, Rotkreuz, Switzerland), LightCycler^®^ DNA Multiplex Master Mix (Roche), 0.25 µl of Uracil-DNA Glycosylase (Sigma Aldrich, Switzerland) and LightMix^®^ Modular Helicobacter 23S rDNA primers and probes (TIBMolbiol, Berlin, Germany). We followed the qPCR Mastermix composition guidelines and the amplification protocol provided by TIBMolbiol. The 23S rDNA qPCR was performed on a LightCycler 480-II^®^ (Roche) followed by melting curve analysis.

For qPCR amplification of the *gyrA* gene, primers by Fujimura et al. (2004) were used. In addition, new primers were designed targeting the same primer binding sites, but accommodating sequence variations found in clinical specimens from different locations in Switzerland (customers of CLM Dr Risch group). Four different forward primers, Hp gyrA-f1 5’-CGATGCATGAATTAGGYCTTACT-3’, Hp gyrA-f5 5’-CGATGCATGAATTAGGCCTCACT-3’, Hp gyrA-f6 5’-CGATGCATGAATTAGGGCTTACT-3’, and Hp gyrA-f8 5’-CGATGCATGAATTAGGCCTTACC-3’, and 2 reverse primers, Hp gyrA-r 5’-TTCTTCACTCGCCTTRGTCAT-3’ and Hp gyrA-r4 5’-TTCTTCGCTCGCTTTGGTCAT-3’, were designed. For *gyrA* qPCR amplification, extracted DNA was added to a mixture consisting of Roche PCR-grade water (Roche), LightCycler^®^ 480 SYBR Green I Mastermix (Roche), 0.25 µl of Uracil-DNA Glycosylase (Sigma Aldrich) and each primer at a final concentration of 0.5 µM. *GyrA* qPCR was performed on a LightCycler 480-II^®^ instrument (Roche) with the following cycler settings: 95°C for 10 min and 45 cycles of 95°C for 10 s, 58°C for 10 s and 72°C for 20 s. Melting curve analysis was performed from 60°C to 90°C with continuous detection. The times at different temperatures were according to the recommendations provided on the polymerase package insert. Annealing temperatures (5 different temperatures between 57°C and 66°C) were tested using the Vircell Amplirun *Helicobacter pylori* DNA control (order number MBC049, distributed by Ruwag, Bettlach, Switzerland). A melting curve was performed after qPCR to distinguish specific and unspecific PCR products.

**Figure 1:**
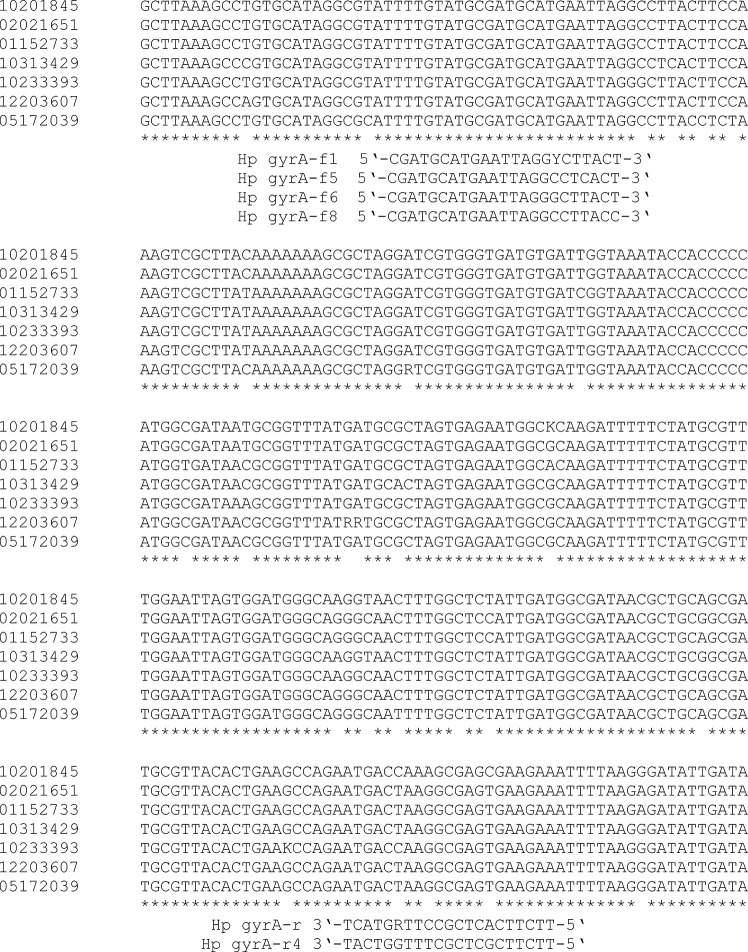
Alignment with ClustalOmega of a fragment of the gyrA gene for the development of new amplification primers. Missing stars indicate sequence variations. The sequences of the two primers “Hp gyrA-f1” and “Hp gyrA-r“ are based on a previous publication (Fujimura et al., 2004). Numbers (left) indicate different gastric biopsies. The reverse primers are shown in this orientation to assign the position of the primers.

To sequence the *gyrA* primer binding sites from clinical specimens with less efficient amplification of *gyrA* than the 23S rDNA, the following primers were used: forward 5’-GGATTGATTCTTCTATTGAAGAGA-3’ and reverse 5’-AAAGGTTAGGCAGACGGCTTGGTA-3’, resulting in a 465 bp long fragment. This 465 bp fragment was sequenced (using the forward primer) and aligned using ClustalOmega. The difference in crossing points (Cp) between *gyrA*-PCR and 23SrDNA-PCR was at least 2.62 with an average of 6.84 (indicating less efficient amplification). The average difference in the Cp-values of the other specimens was less than 1 cycle. The alignment of such sequences is shown below (**Figure 1**). The master mix and cycler settings were equivalent, but the annealing temperature was 55 °C instead of 58°C.

### Genotype HelicoDR Assay

The Genotype HelicoDR assay (Hain Lifesciences GmbH, Nehren, Germany) (Cambau et al., 2009) was performed according to the manufacturer’s recommendations and assessed as interpretable if it showed a positive hybridization signal with the conjugate and amplification control. The assay was assessed as uninterpretable if it showed no hybridization signal with the conjugate control or amplification control. A specimen was reported as positive if the three control bands HP, *gyrA*, and 23S were visible. A band (wild type or mutation) was regarded as positive if the band was at least as intense as the amplification control. However, it is stated that the intensity of the amplification control band might vary depending on the amount of DNA used. There was no exact quantification of the band intensities. Therefore, reporting of the result was dependent on the person involved. Furthermore, the package insert states that the absence of a gyrA87Wt-band or gyrA91Wt-band indicates the presence of resistance. If a mixed infection occurs, at least 2 bands of either codon 87 or codon 91 can be present. The gyrA87MUT band detects N87K but only with AAA as a codon and not AAG. Other mutations at codon 87 are recognizable only by the absence of all gyrA87 bands. Thus, a mixture of a wild-type sequence and a sequence with a mutation other than AAA for codon 87 will to an incorrect report. For codon 91, the HelicoDR test detects D91N, D91G, and D91Y. According to the manufacturer, a single mismatch to a probe leads to loss of the hybridization signal (Hain Lifescience: personal communication).

### Sanger Sequencing of *gyrA* qPCR Products

All *gyrA* qPCR products were sequenced using the four forward primers listed above. PCR products were purified with the HighPure PCR Product Purification Kit (Sigma Aldrich), and Sanger sequencing was performed by Microsynth (Balgach, Switzerland) according to their recommendations. Sequences were aligned using the ClustalOmega online tool (https://www.ebi.ac.uk/Tools/msa/clustalo/).

### Library Preparation and WGS of *H. pylori* Strains

Library preparation was performed using the Qiagen^®^ QIAseq FX DNA Kit (Qiagen, Hilden, Germany) according to the manufacturer’s recommendations. Sequencing library quality and size distribution were analyzed on a fragment analyzer automated CE system (Advanced Analytical Technologies Inc., Heidelberg, Germany) according to the manufacturers’ instructions using the fragment analyzer 474 HS Next Generation Sequencing (NGS) Kit. Sequencing libraries were pooled in equimolar concentrations and paired-end sequenced (2 x150 bp) on an Illumina MiSeq platform (Illumina^®^, San Diego CA, USA).

Raw sequencing reads (fastq) were filtered and trimmed using the FASTQ trimmer tool of the FASTX-Toolkit (Hannon Laboratory, Cold Spring Harbor Laboratories) applying a threshold PHRED score of 25. To identify SNPs in genes conferring resistance, fastq files were analyzed using the ARIBA pipeline (Malfertheiner et al., 2017), querying a custom-made database of gene sequences derived from the *H. pylori* reference strain 26695 (NCBI reference sequence: NC_000915.1).

## Results

### Evaluation of the In-House *gyrA* qPCR as Well as qPCR for 23SrDNA in Comparison With Hybridization-Based HelicoDR Using Gastric Biopsies

*GyrA* qPCR products had a length of 248 bp. The melting temperature (Tm) of the specific products was determined to be between 82.0 and 84.5°C due to the high sequence diversity (**Figure 1**) of clinical specimens (alignment of all positive specimens is not shown). The results were compared to the HelicoDR test, and the results for gastric biopsies described in this publication are those from routine diagnostics of the CLM Dr Risch Group reported to physicians.

DNA from 142 gastric biopsy specimens (104 positive and 38 negative samples) was analyzed by *gyrA* qPCR followed by Sanger sequencing, and the Genotype HelicoDR showed a concordance of 100% regarding positive and negative results. The best match (using NCBIBlast) for Sanger sequencing of *gyrA* was always *H. pylori*, showing high specificity. Regarding the determination of susceptibility or resistance, more differences were observed between the two tests (**Table 2**). Amino acid exchanges leading to resistance were found in 20 specimens with *gyrA* qPCR and Genotype HelicoDR. With qPCR followed by Sanger sequencing, 23 specimens with amino acid exchange leading to resistance were detected. These 23 specimens contained the following mutations: N87I 2 times, N87K 8 times, D91N 5 times, D91G 5 times, and D91Y 3 times. HelicoDR specifically detects only N87K, D91N, D91G, and D91Y. In one clinical specimen, the mutation N87T was detected; however, this mutation is phenotypically susceptible (Lauener et al., 2019). In total, there were 8 discordant results between the two assays (**Table 1**). One of these specimens showed an N87K amino acid exchange in the *gyrA* gene, with an AAG codon identified by Sanger sequencing but no hybridization signal with the Genotype HelicoDR. The lack of a hybridization signal in the Genotype HelicoDR is because the mutation probe for *gyrA* codon 87 detects only the AAA codon for lysine. In addition, two clinical specimens were analyzed as susceptible by the genotype HelicoDR, while amino acid exchanges were found with *gyrA* qPCR combined with Sanger sequencing. The first of these 2 clinical specimens showed a hybridization signal with the gyr87Wt4 probe. However, the signal was clearly weaker than the intensity from the other probes, hinting at inefficient hybridization. *GyrA* qPCR and Sanger sequencing identified an N87I exchange that should not show a hybridization signal with the Genotype HelicoDR. The second specimen clearly showed a D91G exchange with *gyrA* qPCR and Sanger sequencing. The corresponding Genotype HelicoDR assay showed a very weak hybridization signal below the cut-off control and was therefore assessed as susceptible. Notably, there were five specimens that showed weak hybridization signals for a *gyrA* mutation with the Genotype HelicoDR assay (**Table 1**) but did not show a mutation with Sanger sequencing. Since there is no quantitative measurement of the bands of the HelicoDR test and therefore also no quantitative comparison to the control bands, the interpretation of such results remains dependent on the involved person.

**Table 1:** Comparison of qPCR (combined with Sanger-sequencing) and HelicoDR for *gyrA* using gastric biopsies that were positive for *H. pylori*.

		HelicoDR	
		resistant	susceptible
*gyrA* qPCR	resistant	20	3
	susceptible	5	76

**Table 2:** Comparison of qPCR and HelicoDR for 23S rDNA using gastric biopsies that were positive for *H. pylori*.

		HelicoDR	
		resistant	susceptible
23S rDNA qPCR	resistant	26	0
	susceptible	4	74

Additionally, 76 clinical specimens were analyzed as susceptible with both tests.

**Figure 2:**
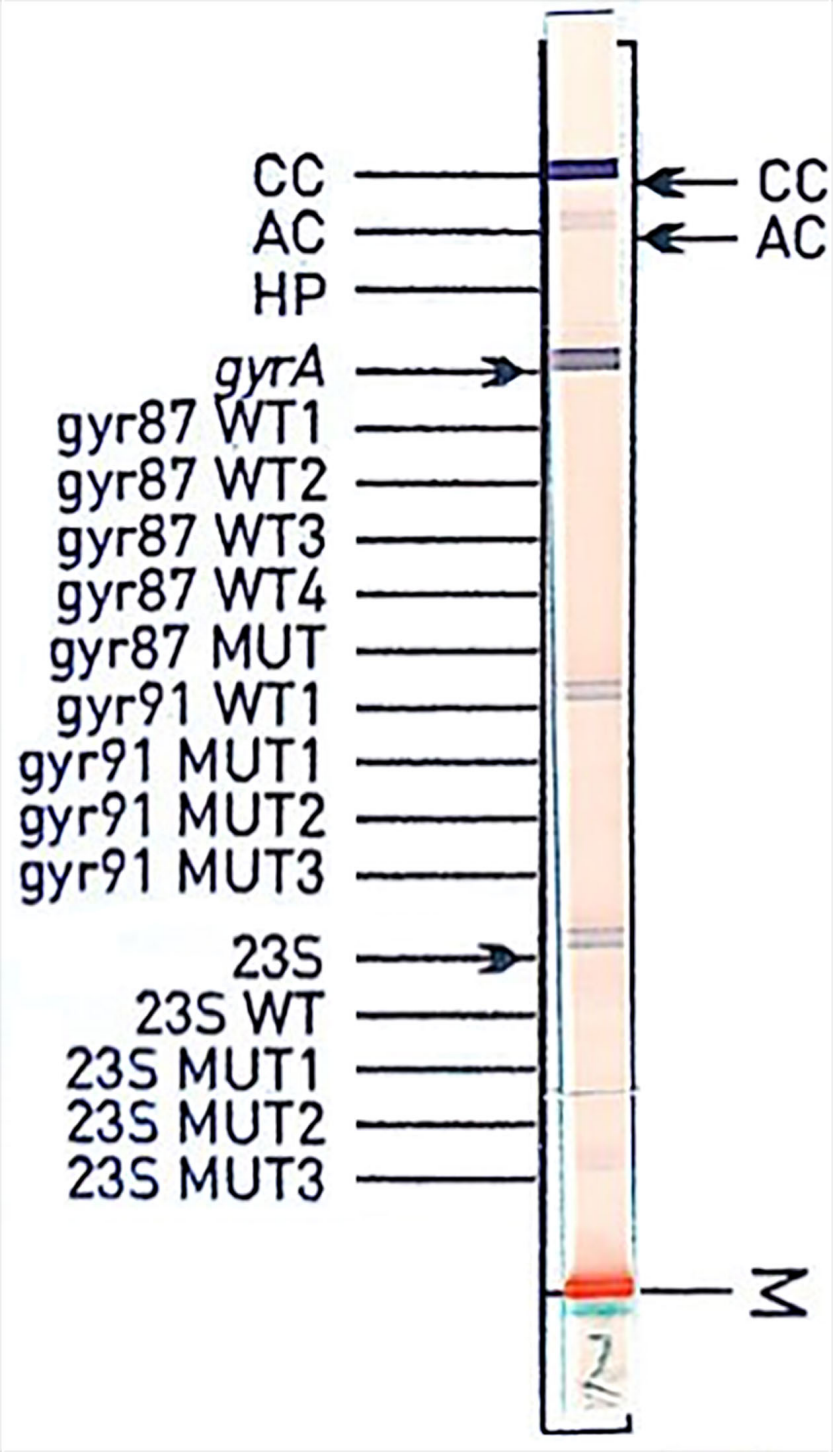
Hybridization assay of specimen from a gastric biopsy. Sanger sequencing shows wild type for both codons 87 and 91 and GCA for codon 88, whereas the most frequent sequence for codon 88 is GCG. The sequence GGCGATAA(C/T)GCAGTTTAT (codons 85 to 90 with AAC or AAT for the most abundant codon 87) with GCA for codon 88 shows a 100% match for only 5 entries with NCBI Blast (accession numbers CP022409.1, CP032043.1, CP032818.1, MK348877.1, CP002332.1: database from August 2019).

Several different (silent) mutations were detected outside codons 87 and 91 with *gyrA* qPCR. An exchange at codon 88 (a GCA instead of a GCG codon) caused no hybridization signal with the gyrA87 probe in the Genotype HelicoDR (see **Figure 2**, example is from different clinical specimens). According to the instruction manual from Hain, samples lacking a hybridization signal with the *gyrA* or 23S rDNA probes should be interpreted as resistant. For this sample, the absence of the gyrA87 amino acid exchange had no implication for levofloxacin resistance prediction, as an additional gyrA91 (D91N) was present. The second specimen equivalently showed an exchange at codon 85 (a GGA instead of a GGC codon) with an additional amino acid exchange at codon 91 (D91N), preventing false resistance prediction. Based on these results, discrepancies must be expected (e.g., mutation only in codons adjacent to codon87 or 91 leading to lack of bands in the hybridization test, which should be interpreted as resistant according to package insert). An additional clinical specimen (not included in the statistics) confirms this statement (**Figure 2**).

Interestingly, there were two clinical specimens with wild-type sequences (Sanger) at codons 87 and 91 but with amino acid changes of E104G or V107I. Phenotypic resistance data were not available for these two specimens. Furthermore, there were 12 specimens with mixed sequences at codons 87 or 91 (determined with qPCR and Sanger sequencing): these were either two different wild-type codons or wild-type and mutated codons. Generally, 24% of gastric biopsies were reported as levofloxacin resistant with the HelicoDR test, and the corresponding value for qPCR and Sanger sequencing was 22%.

Moreover, the agreement between the 23S rDNA qPCR and the Genotype HelicoDR results was assessed and showed a high concordance (26 resistant, 74 susceptible and 4 discrepant) (**Table 2**). With qPCR for 23S rDNA, one clinical specimen was positive for Cp 37 that was reported as negative by HelicoDR. In 23S rDNA qPCR, the 26 clarithromycin-resistant samples included 3 A2146G (11.5%) and 16 A2147G (61.5%) mutations, and the 7 samples with heteroresistance included 1 A2146C and wild type (3.9%), 2 A2146G and wild type (7.7%), and 4 A2147G and wild type (15.4%). The 4 discrepant samples were reported to be susceptible by23S rDNA qPCR and showed very weak hybridization signals in the Genotype HelicoDR. For *gyrA*, the interpretation of week bands is dependent on the experience of the involved person. Generally, 28.8% of gastric biopsies were reported as clarithromycin resistant with the HelicoDR test, and the corresponding value for qPCR was 25%.

Culture and susceptibility results from the 142 gastric biopsies were not available but correlation can be reasonably inferred based upon the data obtained from isolates.

### Determination of Clarithromycin Resistance With 23S rDNA qPCR and WGS Using Cultured Isolates

**Figure 3:**
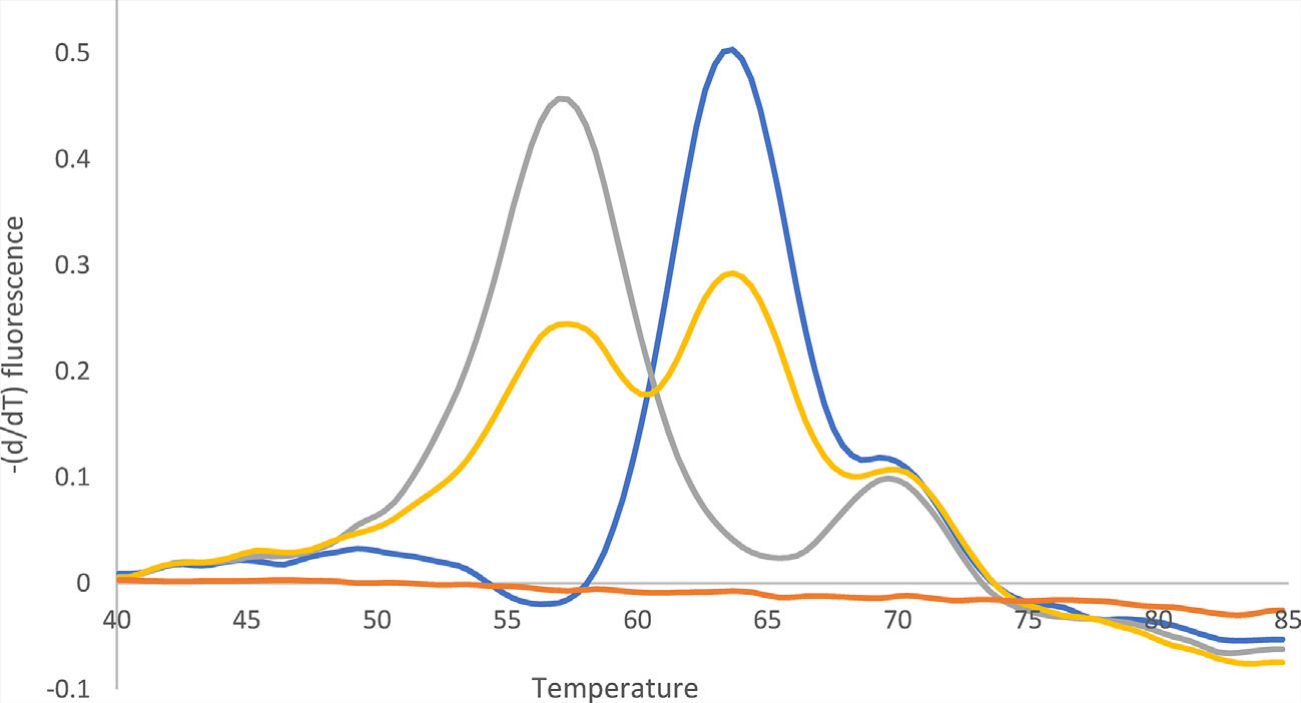
Melting curve after qPCR for 23S rDNA. Yellow: Mixture of A2147G (52%) and wild type (48%). Quantification (%) was performed with WGS. Blue: Wild type. Gray: A2147G. Orange: -K. The peak at 70°C can be ignored (according to the supplier).

**Figure 4:**
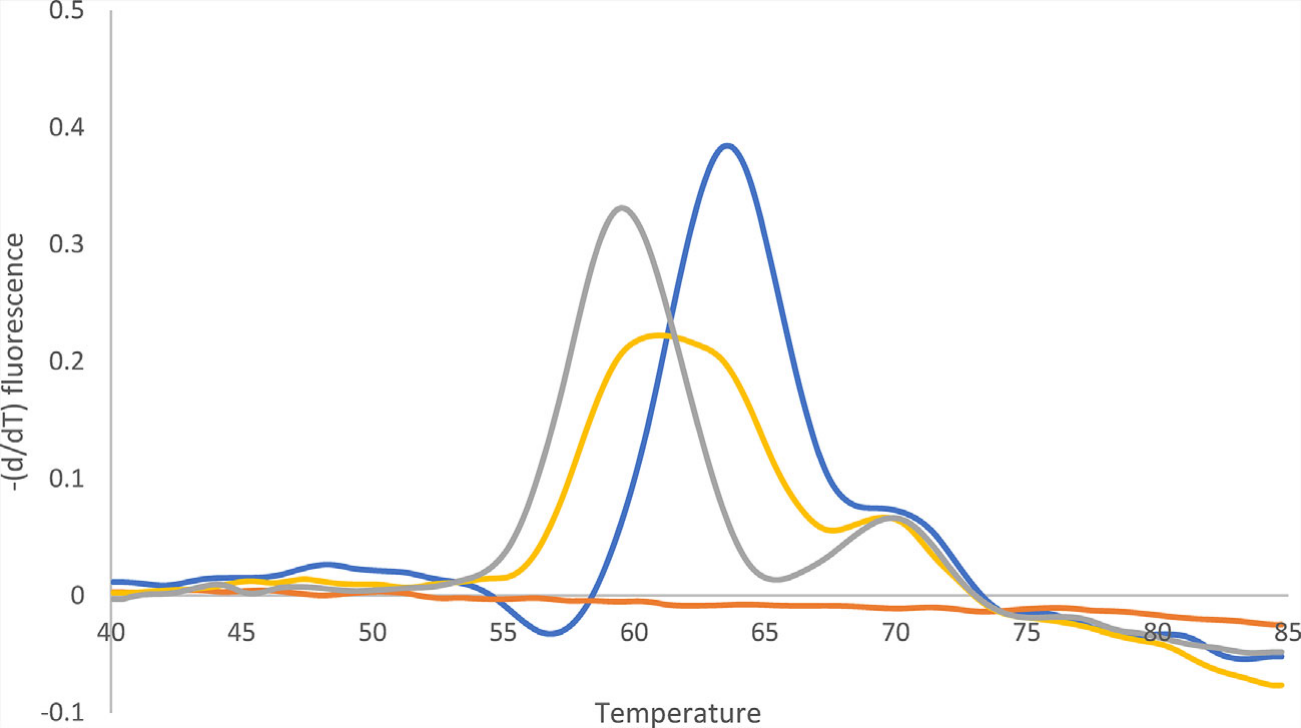
Melting curve after qPCR for 23S rDNA. Yellow: Mixture of A2146G (62%) and wild type (38%). Quantification (%) was performed with WGS. Blue: Wild type. Gray: A2146G. Orange: -K. The peak at 70°C can be ignored (according to the supplier).

Of the 76 *H. pylori* isolates, 49 (64%) were found to be clarithromycin resistant and 27/76 (36%) susceptible by culture-based phenotypic DST. 23S rDNA qPCR detected the following mutations in the 49 clarithromycin-resistant *H. pylori* isolates: 1 A2146C (2%), 12 A2146G (24%), 34 A2147G (70%), and 2 *H. pylori* with heteroresistance (4%; wild type and A2146G and wild type and A2147G) (**Figures 3** and **4**).

WGS identified the following nucleotide exchanges in the 49 *H. pylori* isolates: 1 A2146C (2%), 14 A2146G (31%), and 32 A2147G (67%) (**Table 3**). The two *H. pylori* isolates with heteroresistance identified with 23S rDNA qPCR were confirmed with WGS [one specimen with wild type (38%) and A2146G (62%) and the second specimen with wild type (48%) and A2147G (52%)]. Both *H. pylori* isolates showed clarithromycin MICs of 256 mg/L in culture-based phenotypic DST. As *H. pylori* carries two copies of the 23S rDNA, these two samples may also contain an *H. pylori* gene with heteroresistance (i.e., one wild-type and one mutated 23S rDNA).

**Table 3:** Determination of clarithromycin resistance by phenotypic drug susceptibility testing (DST) 23S rDNA qPCR and WGS in 76 *H. pylori* isolates.

		Culture-based phenotypic DST	
		susceptible	resistant
23S rDNA qPCR	susceptible	27	0
	resistant	0	49
WGS	susceptible	27	0
	resistant	0	49

### Determination of Levofloxacin Resistance With *gyrA* qPCR Combined With Sanger Sequencing and WGS Using Cultured Isolates

**Figure 5:**
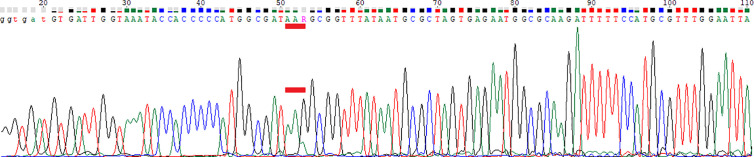
Chromatogram of Sanger sequencing. Red bar marks codon 87. WGS: Position 87, AAG (70%) and AAA (30%). AAA was below the cutoff for WGS (8x detected, cutoff was 10x). AAG and AAA both encode Lys.

Twenty-eight of 76 *H. pylori* isolates (37%) were determined to be levofloxacin resistant and 48/76 (63%) susceptible by culture-based phenotypic DST. Different amino acid exchanges were detected at codons 87 and 91 by *gyrA* qPCR and subsequent Sanger sequencing and WGS (**Tables 4** and **5**). We found mixed sequences at codons 87 and 91 for some *H. pylori* isolates, with both methods (**Table 5**, **Figure 5**). The only discrepant result between *gyrA* qPCR combined with Sanger sequencing and WGS was the mixed *H. pylori* population with wild-type codons 87 and 91 as well as the following 2 amino acid exchanges: N87Y and D91N, where WGS did not identify the D91N exchange. Moreover, one *H. pylori* isolate was determined to be resistant by culture-based phenotypic DST with a levofloxacin MIC of 32 mg/L but did not show an amino acid exchange at codons 87 or 91.

**Table 4:** Determination of levofloxacin resistance by phenotypic drug susceptibility testing (DST), gyrA qPCR combined with Sanger sequencing and WGS in 76 *H. pylori* isolates.

		Culture and phenotypic DST	
		susceptible	resistant
*gyrA* qPCR combined with Sanger sequencing	susceptible	48	1
	resistant	0	27
WGS	susceptible	48	1
	resistant	0	27

**Table 5:** Identified amino acid changes in the *gyrA* gene of 28 levofloxacin-resistant *H. pylori* strains.

Amino acid exchanges	*gyrA* qPCR combined with Sanger sequencing	WGS
N87K and wild type	12	12
N87I and wild type	1	1
N87Y and wild type	–	1
D91N and wild type	3	3
D91G and wild type	4	4
D91Y and wild type	3	3
N87K D91G	2	2
N87K^1^ D91N	1	1
N87K and N87I D91N	1	1
N87Y and wild type D91N and wild type	1	–

^1^Two codons present for lysine: AAG and AAA.

## Discussion

In this study, we aimed to compare different methods for the determination of drug resistance in *H. pylori*. We found very good congruence between the molecular and phenotypic results for cultured isolates.

Phenotypic drug resistance prediction in *H. pylori* is less complex than for other gram-negative bacteria, as drug resistance is based on well-defined point mutations in specific target genes and is not conferred by resistance mechanisms on plasmids or other mobile elements (Lauener et al., 2019).

WGS and qPCR with Sanger sequencing have very good concordance. There are slight differences regarding the sensitivity to mixed sequences, probably because the library preparation for WGS was performed directly from *H. pylori* isolates with only 10 amplification cycles. The sensitivity of mixed sequences is described by Folkvardsen et al. (2013), who reported that 10% of resistance was not detectable with Sanger sequencing. Unfortunately, there is no evidence regarding whether a “low-abundance mutation” leads to phenotypic resistance in *H. pylori*. A previous study (Redondo et al., 2018) showed that there is a high concordance between phenotypical and molecular resistance testing.

Due to high sequence variations in the *gyrA* gene even at positions adjacent to codons 87 and 91, only SYBRGreen PCR followed by Sanger sequencing enables good results for the fast detection of mutations, and new primers must be added to the qPCR if amplification efficiency of *gyrA* qPCR is lower than for 23SrDNA qPCR. Ailloud et al. (2019) and Estibarez et al. (2019) reported that H.pylori is characterized by exceptional high genetic diversity and variability. Our findings regarding the Sanger sequencing of *gyrA* are consistent with this finding. We conclude that a test with a limited set of hybridization probes of approx. 20 bp length (“DNA strip technology” from Hain Lifescience: (Cambau et al., 2009)), which should have a discrimination of 1 bp, is not adequate for the detection of the *gyrA* gene with very high temporal or spatial sequence diversity. This might be the reason why very good commercial assays for the analysis of the gyrA gene are lacking.

The fact that in one phenotypically levofloxacin-resistant *H. pylori* isolate (MIC of 32 mg/L), no mutation in *gyrA* at codon 87 or 91 was detected might be a hint that other resistance mechanisms may also occur. Smith et al. (2014) report that codons 87 and 91 are the most significant mutation sites but not exclusive.

For RT-PCR, the time to result after DNA extraction was less than 1 working day; for 23S rDNA, it was less than 2 h (setup and PCR). The hands-on-time required is much less than for the HelicoDR assay (Hain Lifescience).

Both real-time PCR and the subsequent sequencing of *gyrA*-PCR-positive specimens is cost-effective and fast (**Table 6**). The costs (calculated only for consumables) for both real-time PCR methods decrease with an increased number of specimens per run. Apparently, classical culture remains the cheapest method compared to used molecular methods. However, the obvious advantages of the described molecular methods for direct detection of mutations in gastric biopsies legitimates the higher costs. In contrast, WGS is obviously costlier than qPCRs.

**Table 6:** Comparison of the assay characteristics of phenotypic drug susceptibility testing (DST), qPCR, and melting curve analysis, Sanger sequencing and whole-genome sequencing (WGS).

	*H. pylori* culture and phenotypic DST	qPCR(23S rDNA and *gyrA*)	Sanger sequencing(*gyrA* from qPCR)	WGS
Starting material	clinical specimen	clinical specimen	–	*H. pylori* culture
Time to result	7 to 14 days	<4 h	1 day	2 days
Costs (EUR)	20.-	5.- DNA isolation max 20.- qPCR	20.-	150.-

For both qPCR methods, costs are reduced for larger series. Costs (EUR) are per sample (culture or biopsy).

Due to an increasing trend towards high primary and secondary levofloxacin resistance in *H. pylori* (Zullo et al., 2007; Boyanova et al., 2010; Cuadrado-Lavín et al., 2012; Saracino et al., 2012), it is encouraged to analyze the *gyrA* gene in addition to performing qPCR for 23S rDNA. This recommendation is supported by the results of gastric biopsies (direct detection) with 22%–24% resistance for levofloxacin and 25%–29% for clarithromycin (the range is due to the slight difference between the two test procedures).

Since the *gyrA* gene is a single-copy gene (Tomb et al., 1997), mixed sequences are always mixed infections. Sanger sequencing showed were 4 mixed infections (5.2%) among the 76 cultured isolates based on the sequence diversity of the *gyrA* gene.

A review article from African studies (Jaka et al., 2018) declares a very broad percentage of clarithromycin resistance (between 0% and 100%) with an overall resistance of 29.2%. Additionally, this review declares a quinolone resistance of 0% to 32% with the overall resistance of 17.4% (Jaka et al., 2018). Another review (Savoldi et al., 2018) declares in European region an overall clarithromycin resistance of 32% and levofloxacin resistance of 14%. Another study (Megraud et al., 2013) with data in Europe declares a clarithromycin resistance of 17.5% and levofloxacin resistance of 14.1%, however with generally lower percentages in Northern European countries. Our data show, that the clarithromycin resistance, which was detected directly from gastric biopsies, remains high as previously found in one of the two reviews. However, for levofloxacin resistance (direct detection from gastric biopsies as well), our data shows an increased percentage of levofloxacin resistant *H. pylori* infections. Since the usage of qPCRs for 23SrDNA are prevailing (i.e., without analysis of *gyrA* (Bénéjat et al., 2018)), it can be suspected that prescription of levofloxacin for those numerous cases with detected clarithromycin resistance might select *gyrA* mutations (Marques et al., 2020). qPCRs are proven to be of clinical relevance to avoid difficulties of culture (Bénéjat et al., 2018). The parallel testing (qPCR for 23SrDNA and *gyrA*) enables the opportunity for adequate antibiotic treatment, since also other antibiotic classes can be used for treatment (Lauener et al., 2019) even for those cases with dual resistance (clarithromycin and levofloxacin).

## Conclusion

Since the successful isolation and cultivation of *H. pylori* from gastric biopsy specimens is a challenging task hampered by a number of technical factors and is long-lasting as well, it is beneficial for the timely treatment of the patient to have an adequate molecular test which enables resistance testing directly from gastric biopsies. Application of qPCR is meanwhile well-established and results therefore in novel diagnostic tools. In particular, the novel qPCR (23SrDNA and *gyrA*) and (Sanger) sequencing (*gyrA*) can overcome disadvantages of cultures and should be applied for the detection of *H. pylori* in gastric biopsies. The two evaluated qPCR tests for 23S rDNA and *gyrA* are much faster than culture, sensitive, specific, include carry-over prevention and were extensively, which includes cultured isolates as well as direct detection from gastric biopsies. Due to the high percentage of both levofloxacin and clarithromycin resistance, which was directly detected in gastric biopsies (both between 22% and 29%), both targets have to be analyzed in parallel. Furthermore, due to high sequence variations in the *gyrA* gene, SYBRGreen PCR followed by Sanger sequencing is currently the best and fastest available molecular method. WGS strongly supports results of both qPCR using cultured isolates. For the direct detection of *H. pylori* in gastric biopsies, WGS has the limitation that the strong abundance of human DNA might reduce sensitivity of the two relevant targets.

## Data Availability Statement

The datasets generated for this study can be found in NCBI GenBank, NCBI Accession No.’s MW057345-51. The sequence data from WGS are listed in Lauener FN, Imkamp F, Lehours P, Buissonnière A, Benejat L, Zbinden R, Keller PM, Wagner K (2019) Genetic determinants and prediction of antibiotic resistance phenotypes in Helicobacter pylori. J Clin Med 8:53. doi:10.3390/jcm8010053.

## Author Contributions

KE: conceptualization, methodology, validation, investigation, resources, and writing. KW: methodology, validation, resources, and writing. PK: resources, review, and editing. LR: funding acquisition. MR: funding acquisition. TB: project administration and conceptualization. All authors contributed to the article and approved the submitted version.

## Funding

This scientific work is funded by the affiliations mentioned for the authors, mainly by “labormedizinisches zentrum Dr Risch”.

## Conflict of Interest

The authors declare that the research was conducted in the absence of any commercial or financial relationships that could be construed as a potential conflict of interest.
